# A pilot open label, single dose trial of fenobam in adults with fragile X syndrome

**DOI:** 10.1136/jmg.2008.063701

**Published:** 2009-01-06

**Authors:** E Berry-Kravis, D Hessl, S Coffey, C Hervey, A Schneider, J Yuhas, J Hutchison, M Snape, M Tranfaglia, D V Nguyen, R Hagerman

**Affiliations:** 1 Departments of Pediatrics, Neurological Sciences, Biochemistry, Rush University Medical Center, Chicago, Illinois, USA; 2 Department of Psychiatry and Behavioral Sciences, University of California Davis, Davis, California, USA; 3 MIND Institute, University of California Davis Medical Center, Davis, California, USA; 4 Department of Pediatrics, Rush University Medical Center, Chicago, Illinois, USA; 5 Neuropharm Ltd, Leatherhead, Surrey, UK; 6 FRAXA Research Foundation, Newburyport, Massachusetts, USA; 7 Department of Public Health Sciences, University of California Davis, Davis, California, USA; 8 Department of Pediatrics, University of California Davis Medical Center, Davis, California, USA

## Abstract

**Objective:**

A pilot open label, single dose trial of fenobam, an mGluR5 antagonist, was conducted to provide an initial evaluation of safety and pharmacokinetics in adult males and females with fragile X syndrome (FXS).

**Methods:**

Twelve subjects, recruited from two fragile X clinics, received a single oral dose of 50–150 mg of fenobam. Blood for pharmacokinetic testing, vital signs and side effect screening was obtained at baseline and numerous time points for 6 h after dosing. Outcome measures included prepulse inhibition (PPI) and a continuous performance test (CPT) obtained before and after dosing to explore the effects of fenobam on core phenotypic measures of sensory gating, attention and inhibition.

**Results:**

There were no significant adverse reactions to fenobam administration. Pharmacokinetic analysis showed that fenobam concentrations were dose dependent but variable, with mean (SEM) peak values of 39.7 (18.4) ng/ml at 180 min after the 150 mg dose. PPI met a response criterion of an improvement of at least 20% over baseline in 6 of 12 individuals (4/6 males and 2/6 females). The CPT did not display improvement with treatment due to ceiling effects.

**Conclusions:**

Clinically significant adverse effects were not identified in this study of single dose fenobam across the range of dosages utilised. The positive effects seen in animal models of FXS treated with fenobam or other mGluR5 antagonists, the apparent lack of clinically significant adverse effects, and the potential beneficial clinical effects seen in this pilot trial support further study of the compound in adults with FXS.

Fragile X syndrome (FXS) is the most common inherited form of intellectual disability, autism, and learning disability, with a broad range of severity and full mutation gene frequency of 1/2500.^1^ FXS results from an unstable trinucleotide repeat expansion of >200 CGG repeats (full mutation) in the promoter of the *FMR1* (fragile X mental retardation–1) gene^2^ which leads to transcriptional silencing of *FMR1* and thus, absence or significant reduction of the *FMR1* protein (FMRP).^3^ Because *FMR1* is located on the X chromosome, females with a full mutation are more mildly affected than males, due to production of FMRP from the normal *FMR1* allele on the non-mutated X chromosome. FMRP is an RNA binding protein which modulates dendritic maturation and synaptic plasticity through mechanisms including inhibition of group 1 metabotropic glutamate receptor (mGluR1 and mGluR5) mediated mRNA translation in dendrites.^4^^–^7 Numerous expected consequences of excessive activation of mGluR mediated dendritic protein synthesis due to loss of inhibitory control by FMRP are found in the *fmr1* knockout mouse, including enhanced mGluR activated hippocampal^8^ and cerebellar^9^ long term depression (LTD), reduction of synaptic AMPA receptors,^10^ immature appearing elongated dendritic processes,^11^ ^12^ and abnormal epileptiform discharges.^13^ Further, many phenotypic features of FXS are predicted effects that would occur in a setting of enhancement of mGluR mediated processes, including seizures, epileptic abnormalities on electroencephalograms (EEGs), cognitive problems, strabismus, enhanced anxiety, perseverative behaviours, coordination problems, hypersensitivity to tactile stimuli, and even loose stools.^10^

Consistent with this underlying mechanism of mGluR overactivity in FXS, MPEP (2-methyl-6-(phenylethynyl)-pyridine) and other mGluR negative modulators have been shown to reverse multiple phenotypes in the *fmr1* knockout mouse, including audiogenic seizures, epileptiform discharges and open field hyperactivity,^13^ ^14^ as well as impairments in courtship memory in *dfxr* mutant *Drosophila*^15^ models. Genetic down regulation of mGluR5 expression by crossing the *fmr1* KO mouse with mGluR5 heterozygous knockouts^16^ also reverses these and other phenotypes including dendritic spine changes, ocular dominance plasticity, and excessive protein synthesis.

Although mGluR5 negative modulators are not currently available for treatment of humans with FXS, during recent high throughput lead-finding screens, Porter *et al*^17^ discovered that fenobam is a high potency and highly selective mGluR5 antagonist, comparable to MPEP, with no relevant effects on a panel of 86 central nervous system (CNS) receptors assayed in a commercial receptor binding screen, including other mGluRs. Fenobam was previously investigated as an anxiolytic in a number of phase II studies in the early 1980s.^18^^–^20 These studies revealed a mixed picture of anxiolytic efficacy, with double blind, placebo controlled trials variously reporting the compound as active or inactive. This discrepancy was not easily reconciled based on patient numbers, dose level, duration of treatment, or outcome measures. There were no major safety concerns although a number of subjects taking doses of 150 mg four times daily of fenobam for up to 4 weeks did describe odd CNS related perceptual phenomena, such as hallucinations, vertigo, paraethesias, and insomnia.

Given the likelihood that fenobam would target a specific underlying mechanism of neural dysfunction in FXS, we initiated an open label, single dose study to provide an initial evaluation of the safety and pharmacokinetics of fenobam in adult males and females with FXS, and to explore effects of fenobam on core phenotypic measures of sensory gating, attention and inhibition in FXS. The CNS side effects previously reported in clinical studies with fenobam have the potential to be subjectively distressing. FXS represents a vulnerable patient population with impaired understanding and communication skills. Negative modulators of the mGluR5 receptor are capable of producing morphological and behavioural changes after a single dose.^21^ ^22^ Accordingly we felt it most appropriate to examine the effects of fenobam in patients with FXS with a single dose in this first clinical trial of an mGluR5 antagonist.

## METHODS

Subjects were recruited from fragile X clinics at Rush University Medical Center (RUMC) and the MIND Institute at University of California at Davis Medical Center (UCDMC). Inclusion criteria required a DNA based diagnosis of FXS, stable medication doses for at least 6 weeks before study, and ability to tolerate an intravenous catheter (IV) for 6 h for pharmacokinetic studies. Exclusion criteria included concurrent treatment with lithium, typical antipsychotics, tricyclic antidepressants, NMDA antagonists or enzyme inducing anticonvulsants, concurrent or recent initiation of cognitive behavioural therapy, significant disease in another organ system, hearing or vision impairments, psychosis, major depressive symptoms, pregnancy, drug abuse disorder, Tourette syndrome, and significant abnormalities in baseline laboratory tests. Subjects with well controlled seizures were not excluded although none of the subjects enrolled had a seizure history. Informed written consent was obtained from either the subject or the parent before participation. Assent from the subject was obtained when the subject was not his/her own legal guardian. The study was approved by the institutional review boards at RUMC and UCDMC. The sequence of subject enrolment and treatment was random, depending only on when subjects contacted the study centre and when they could be scheduled.

At the screening visit medical history, exam, vital signs, laboratory testing including routine chemistries, blood counts, thyroid functions, electrocardiogram (ECG), and a pregnancy test (females) were evaluated, and baseline CPT (Carolina Project Fragile X continuous performance test) and prepulse inhibition (PPI) outcome measures were obtained. At the treatment visit, 14–28 days after screening, an IV was inserted for blood drawing and the subject received the study medication orally. Blood for pharmacokinetic testing, blood pressure, heart rate and side effect screening was obtained at 0, 15, 30, 45, 60, 120, 180, 240, 300, and 360 min after dosing. Our side effects screening protocol involved asking both the subject and family members if the subject was having any of a structured list of symptoms relevant to potential effects of fenobam (aggression, fatigue, hyperactivity, anxiety and fidgetiness, increase in self stimulation, odd behaviour, inappropriate laughter, dizziness, vertigo, nausea, paraesthesia, and headache), and direct observation by the physician and family for the above symptoms and other behavioural changes. Subjects were all verbal and sufficiently high functioning to report side effects in response to simple questions. Post-treatment PPI was performed after the 60 min blood sampling, followed by CPT. The first subject for each gender was dosed with 50 mg fenobam, the second with 100 mg, and all subsequent subjects with 150 mg. The final dosage of 150 mg fenobam was at the middle range of individual doses in previous studies where CNS side effects were observed. Conference calls were held after each subject was dosed, to confirm absence of adverse events and justify dose escalation or maintenance for the subsequent subject of that gender.

PPI was chosen as an outcome measure to assess sensorimotor gating and inhibitory control because: (1) there is significant deficit of PPI in males and females with FXS^23^ ^24^; (2) PPI at 120 ms has excellent test-retest reliability with intraclass correlations of 0.85 for FXS and 0.88 for controls^24^; (3) PPI is responsive to medication effects^25^ ^26^; (4) PPI in the mouse model of FXS can be corrected with MPEP^21^; and (5) PPI represents an electrophysiological measure that is expected to be less amenable to placebo effects than other measures. The PPI protocol used for this study was a slightly modified version of the procedure previously described.^24^ Startle stimuli (SS) were 50 ms 105 db SPL white noise pulses and acoustic prepulses (PP) are 25 ms 75 db SPL 1 kHz tones. These trials are delivered while participants watch a silent movie to maintain compliance and interest in the procedure. Test-retest studies have demonstrated good to excellent reliability for PPI at 120 ms and 240 ms, but inadequate reliability for PPI at 60 ms. Therefore, for the current study, we eliminated the 60 ms trial type and added two trials per type, resulting in a protocol with 30 total trials (10 with SS alone, 10 with 120 ms prepulse and 10 with 240 ms prepulse). Digitised obicularis oculi electromyographic peak magnitudes recorded between 20 ms and 200 ms post startle probe onset were averaged across trials within each type. PPI was calculated as: 100 × [(mean response magnitude in the startle stimulus alone trials – mean response magnitude in the prepulse trials)/mean response magnitude in the startle stimulus alone trials]. Based on group differences obtained in our prior study and review of the literature on PPI reliability and its response to psychopharmacological intervention, we determined a priori that subjects with PPI improvement of 20% on the 120 ms trials (the most reliable trial latency) or more would be considered responders.

The Carolina Fragile X Project Continuous Performance Test (FXCPT), developed for individuals with cognitive impairment, was chosen to assess attention, impulsivity, and inhibition, as other more standard CPT measures are too difficult for individuals with FXS.^27^ The FXCPT showed a significant deficit in response inhibition in males with FXS, compared to mental age matched controls,^28^ and in a test-retest reliability study had a weighted kappa of 0.7, suggesting a good reproducibility.^29^ The FXCPT was administered as previously described^28^ ^29^ and the number of correct hits on a target stimuli, omissions involving lack of reaction to a target (attention deficit), and commissions involving reacting to a non-target (impulsivity, poor inhibitory control) were tabulated. Individuals with FXS often hit the mouse button repeatedly and impulsively, thus achieving a perfect number of hits with low omission scores, but a poor overall performance due to a high number of commission errors.^29^ Thus, the commission score was the main focus of the analysis, as the more reliable marker for abnormal performance on the FXCPT.

Fenobam concentrations from plasma samples were measured with MS-MS based assays validated for human application, using positive ion liquid chromatography/mass spectrometry/mass spectrometry (LC/MS/MS). For comparison, plasma samples over 24 h were obtained from three healthy adult male volunteers.

For statistical analysis of PPI data, a positive response was defined as a 20% or greater improvement over baseline. One sample exact binomial 95% confidence interval was computed for the proportion of positive response. A one sample two sided exact hypothesis test was used to compare the proportion of positive response to the null proportion of 2/13 (15.4%), obtained from our prior PPI study with untreated individuals with FXS.^24^

## RESULTS

Six males and six females with FXS (three males and three females at each site; mean (SD) age 23.9 (5.4) years, range 18.7–30.7 years) were screened and enrolled. There was wide variability in cognitive ability, with IQs ranging from 36–85, as would be expected in a study enrolling both males and females with FXS. No subjects dropped out or failed to meet entry criteria. There were no clinically significant abnormalities of the examination, vital signs, or laboratory or ECG parameters at baseline. Demographic data are given in table 1.

**Table 1:** Characteristics and responses of fragile X syndrome subjects treated with single dose fenobam

Study*	Gender	Ethnicity	IQ	Concomitant medications†	Fenobam dose (mg)	Side effects‡	PPI§	Type of improvement noted clinically‡
01-001	M	C	53	Aripiprazole quetiapine	50	None	+	Improved eye contact and interaction
01-002	M	C	55	Venlafaxine aripiprazole	150	None	+	Improved interaction
01-003	F	AA/H	85	Venlafaxine	150	Mild sedation	–	No improvement
01-004	M	C	52	None	150	Mild sedation	–	Calmed behaviour, improved eye contact, less perseveration
01-005	F	C	71	None	150	None	+	Improved interaction
01-006	F	C	66	None	150	Mild sedation	–	Calmed behaviour, less nervous giggling, improved eye contact
02-001	M	C	N/A	None	100	None	–	Improved eye contact
02-002	F	C	54	Dextro-amphetamine fluoxetine	50	None	+	Calmed behaviour and improved eye contact
02-003	F	C	53	Methylphenidate	100	None	+	No improvement
02-004	M	C	36	Fluoxetine	150	None	+	Improved eye contact and interaction
02-005	M	AA	36	Dextro-amphetamine	150	None	–	No improvement
02-006	F	C	50	Escitalopram	150	Anxious, tremulous, clammy. Lightheaded when IV was put in	–	Calmed behaviour, able to tolerate blood draws better, much more tolerant of IV placement
Average (SD)	55.5 (13.6)							
range	36–85							

AA, African American; C, Caucasian; F, female; H, Hispanic; IV, intravenous catheter; M, male; N/A, IQ measurements were not available for this subject.

*01 = MIND Institute UCDMC; 02 = Rush University Medical Center.

†Only psychoactive concomitant medications; 3 subjects on stimulants, 3 on selective serotonin reuptake inhibitors, 2 on other antidepressant, 2 on atypical antipsychotics.

‡Side effects and clinical improvement were characterised by observations at designated time points (0,15, 30, 45, 60, 120, 180, 240, 300, 360 min) by the principal investigator (PI) and by formal questioning of the subject/guardian throughout the visit for the occurrence of central nervous system (CNS) side effects relevant to fenobam from a checklist (see Methods) new, or worsening of existing signs, symptoms or behaviours.

§Result of prepulse inhibition (PPI) after fenobam relative to baseline; “+” denotes achieved response criterion of 20% improvement in PPI and “–” represents <20% improvement.

There were no significant adverse reactions to fenobam. Particularly, adverse events (listed in Methods) in relation to altered CNS function were not seen. Three subjects experienced mild sedation (table 1). In nine of the 12 cases calmed behaviour was observed with improvement in eye contact, ability to interact, anxiety and/or motor overactivity (table 1).

**Figure 1:**
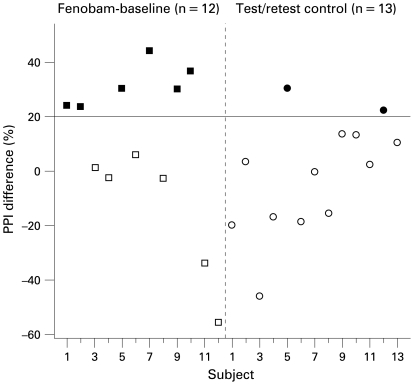
Change in prepulse inhibition (PPI) in adults with fragile X syndrome (FXS) after single dose fenobam compared to variation in a prior control group with FXS (18.70 (9.56) years) that underwent test-retest with a similar PPI protocol performed at the same study sites over the same time frame.^24^ Four of six males (67%) and two of six females (33%) had PPI increases of 20% or more (improvement criterion) after fenobam compared to two of 13 with FXS (15%) in the test-retest control group.

PPI for the 120 ms trials met response criterion of at least 20% improvement over baseline in six of 12 individuals (four of six males and two of six females, table 1) with 95% confidence interval of 21.1% to 78.9%. Improvements ranged from 23.7% to 44.2% (fig 1). The proportion of positive response (at least 20% improvement after fenobam) was significantly greater than that expected in untreated individuals (p = 0.01), which was found to be 15.4% (2/13) based on a prior study^24^ consisting of a similar cohort of individuals with FXS who underwent test-retest on the PPI utilising the same methodology in the absence of intervention (fig 1).

As expected, most subjects (10 on auditory FXCPT, nine on visual FXCPT) had a 100% rate of hits at baseline. The rate of commissions was also low, with only one subject on auditory FXCPT and one on the auditory and visual FXCPT showing more than two commission errors at baseline. Thus, there were no significant changes between baseline and treatment in FXCPT performance due to ceiling effects. However, the most impaired subject on this task did show a decrease in commissions (from 18 to 12 on visual FXCPT and 26 to six on auditory FXCPT).

**Figure 2:**
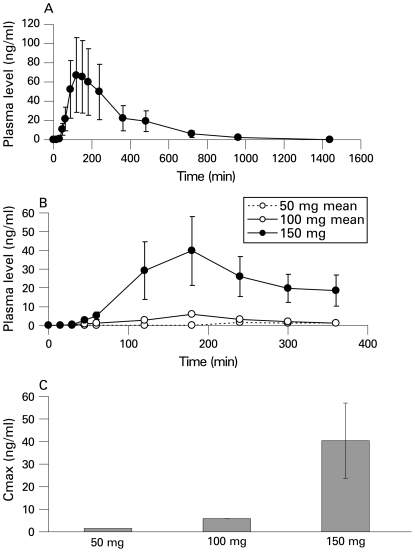
Plasma concentrations of fenobam following oral administration. Panel A shows plasma values in n = 3 male healthy adult volunteers administered a single dose of 150 mg fenobam monohydrate. The mean (SEM) plasma concentration achieved was 67.1 (37.8) ng/ml at 120 min post dose. Healthy volunteers were tested through a different phase I pharmacokinetic study of the formulation of fenobam to be used in the fragile X syndrome (FXS) study. These individuals were tested at Qualia Clinical Services, Inc, Nebraska, USA, during 2007 after they signed informed consent. The protocol for pharmacokinetic testing in the volunteers was approved by the Qualia Clinical Services Inc independent institutional review board (IRB) and submitted to the US Food and Drug Administration. Time points for collection of the pharmacokinetic data were slightly different from those used in the subjects with FXS. Panel B shows plasma values of fenobam following administration of 50 mg (n = 2), 100 mg (n = 2) and 150 mg (n = 8) to male and female adults with FXS. Samples were obtained from patients during the duration of a single outpatient visit. Following administration of 150 mg fenobam to patients the mean (SEM) plasma concentration achieved was 39.7 (18.4) ng/ml at 180 min post dose. The difference between mean peak values after administration of 150 mg fenobam was not significant when normal healthy adults males were compared to subjects with FXS (t = 0.47, df = 2, p = 0.67). The mean peak plasma values obtained after administration of 150 mg fenobam to males and females with FXS were also not significant (t = 0.28, df = 6, p = 0.79). Panel C shows mean (SEM) plasma values achieved following administration of 50 mg (n = 2), 100 mg (n = 2) and 150 mg (n = 8) fenobam monohydrate in male and female adult patients with FXS. The mean peak plasma value achieved was related to dose.

Pharmacokinetic analysis in subjects with FXS showed that fenobam values were dose dependent but variable, with mean (SEM) peak concentrations following administration of 150 mg of fenobam being 39.7 (18.4) ng/ml at 180 min post dose. The mean peak plasma concentration of fenobam did not differ from that seen in normal volunteers (n = 3). Upon visual inspection the timing to mean peak concentrations was similar in FXS patients and in normal controls (fig 2). Neither fenobam dose nor concentrations after 60 or 120 min correlated with improvement in PPI.

## DISCUSSION

To our knowledge this is the first study assessing safety and pharmacokinetic metabolism of an mGluR5 antagonist in humans with FXS. Administration of a single dose to this cohort of 12 adults did not result in significant adverse events. Although the doses of 100 mg to 150 mg are within the range (100–600 mg) of single fenobam doses previously investigated in patients with anxiety disorders, and despite extensive questioning of subjects and parents, side effects described in previous studies were not observed. The pharmacokinetics of fenobam showed wide intersubject variability, as previously noted in normal volunteers and patients with anxiety disorders. Peak plasma concentrations in this study ranged from 3.28–113 ng/ml (5.3–220 ng/ml in prior studies^20^) which overlays the concentration range at which fenobam interacts with the mGluR5 receptor.^17^ Peak plasma times occurring around 2–3 h were slightly longer than reported previously (0.33–2 h).^20^

In a subset of subjects, calmed behaviour was observed within an hour after fenobam dosing, before the mean peak plasma concentration of fenobam. The observation of rapid reduction in hyperactivity and anxiety after fenobam dosing in the most affected subjects with FXS was particularly surprising. Similarly, it was remarkable that 50% of our cohort demonstrated at least a 20% improvement in PPI whereas only 15.4% (two of 13) of untreated individuals with FXS and 0% of normal controls (0 of 16) had 20% or more increase in PPI in test-retest studies. PPI improved in subjects treated with 50, 100, or 150 mg of fenobam (table 1), consistent with variable pharmacokinetics of the drug and suggesting that, as with many psychoactive drugs, effective dosing may be highly variable in different individuals. Similarly, clinical responsiveness was variable and not specific to the dosage used in this study. Five of the six individuals with improved PPI also showed a subjective clinical improvement and one did not, and four individuals showed clinical improvement but not an improved PPI, so there was not complete concordance between PPI and clinical improvement. However, PPI was only done at 1 h post dosing, when fenobam values had not yet peaked, and clinical assessment was continuous for 6 h after the dosing; therefore, clinical observations were likely more sensitive, given the pharmacokinetic variability of fenobam, but the PPI data are less subject to placebo effect and thus provide a useful additional indicator of response.

The rapid improvement in PPI is consistent with prior studies suggesting that PPI can respond acutely to drug treatments; children with ADHD showed improvement in PPI after a single dose of methylphenidate,^25^ and mGluR agonists produce rapid (within 30 min of treatment) normalisation of PPI in the knockout mouse model of FXS.^21^ Thus, although more permanent synaptic reorganisation associated with cognitive improvement would be expected to take much longer, PPI may be at least partially dependent on rapid changes in neurotransmitter and neuroreceptor expression modulating short term synaptic plasticity.

Fenobam peak concentrations occurred later than expected based on prior pharmacokinetic results and thus the PPI was done when values were still increasing on the pharmacokinetic curve. Therefore, the PPI results presented may represent an underestimate of the effect of fenobam on PPI that would have been measured if PPI was done at 2–3 h post-dose. Further, correlations between PPI and fenobam values may have been masked due to variable rates of increase in different subjects. In future study design for fenobam trials, PPI should be administered 2–3 h post-dose to optimise drug impact. The PPI results shown here are consistent with a recent study using eyeblink startle PPI methodology, similar to methods used in human studies, which demonstrated a significant PPI deficit in the fmr1 knockout mouse that was rescued to wild-type levels by MPEP, an mGluR5 antagonist.^21^

The poor correlation of PPI improvement with fenobam values, and the observation of behavioural and PPI responses well before the peak level of fenobam was reached, could be due to rapid generation of an active metabolite with a variable rate of conversion or because the observed change was unrelated to fenobam. As expected, PPI results for females are more variable than males, likely due to neuronal and brain circuit mosaicism for FMRP production. Thus, depending on brain patterns of FMRP mosaicism, PPI deficits (and rescue) would not necessarily be correlated with cognition in females with FXS.

Although the FXCPT did not show significant changes from baseline to treatment conditions in the averaged group results, some individual improvements in commission errors were observed. This CPT is not sufficiently sensitive to be used as an outcome measure in relatively high functioning cohorts of subjects with FXS due to ceiling effects. Additional work is needed to identify a CPT that functions as a measure of attention and inhibition across the entire spectrum of involvement in FXS as well as other outcome measures, such as a validated FXS specific scale directed at measuring change in behavioural phenotypes prominent in FXS.

Limitations of this study include the lack of placebo control, the small sample size, and the biasing of the subject sample to include only those who were higher functioning behaviourally and less anxious so that they would be able to tolerate the study procedures, including the intravenous catheter. More anxious individuals with FXS might be expected to show a more obvious response to the drug, but would not have tolerated this protocol. Also, there may have been limitations in the ability of subjects to describe side effects despite the extensive measures taken to elicit these, and the study does not rule out side effects that might emerge with chronic dosing, as in cohorts treated with fenobam in the 1980s. Nonetheless, the lack of safety problems seen in this study should encourage further studies of fenobam, starting at low doses and with careful titration based on efficacy and tolerability, in patients with FXS—given the remarkable benefits of mGluR5 negative modulators with respect to behaviour, cognition and even dendritic structure in animal models of FXS. It is hoped that long term use of fenobam will similarly rescue synaptic plasticity deficits in humans with FXS, and that PPI improvement observed here signals enhanced frontal gating, which with long term treatment could lead to improved information processing and cognition. To effect long term improvements in cognition, pharmacological treatment will likely require concomitant learning programmes to provide the substrate for the stimulus dependent synaptic remodelling presumably facilitated by the mGluR5 blocker. This will necessitate re-initiation of learning programmes for adults and emphasises the need to work toward trials of fenobam in children, who are still in school, and are earlier in the course of the effects of FXS on synaptic plasticity.

In summary, this trial did not find major safety concerns to a single administration of fenobam in FXS, and suggested that clinical improvements in behaviour and PPI may be seen even after a single dose. This would indicate that placebo controlled trials of fenobam and other mGluR5 antagonists involving longer term treatment of individuals with FXS should be considered to investigate whether rescue of the FXS phenotype observed in animal models can be extended to humans.
